# The research landscape of ferroptosis in neurodegenerative disease: a bibliometric analysis

**DOI:** 10.3389/fnagi.2024.1417989

**Published:** 2024-06-17

**Authors:** Yun Liu, Dan Feng, Ling Shui, Yu-jie Wang, Li Yu, Yu-qi Liu, Jin-yong Tian

**Affiliations:** ^1^First Clinical Medical College, Guizhou University of Traditional Chinese Medicine, Guiyang, China; ^2^Department of General Practice, Guizhou Provincial People's Hospital, Guiyang, China

**Keywords:** ferroptosis, neurodegenerative diseases, bibliometric analysis, hotspots, CiteSpace, knowledge graph

## Abstract

**Background:**

Ferroptosis, a newly proposed concept of programmed cell death, has garnered significant attention in research across different diseases in the last decade. Despite thorough citation analyses in neuroscience, there is a scarcity of information on ferroptosis research specifically related to neurodegenerative diseases.

**Method:**

The Web of Science Core Collection database retrieved relevant articles and reviews. Data on publications, countries, institutions, authors, journals, citations, and keywords in the included studies were systematically analyzed using Microsoft Excel 2019 and CiteSpace 6.2.R7 software.

**Result:**

A comprehensive analysis and visualization of 563 research papers on ferroptosis in neurodegenerative diseases from 2014 to 2023 revealed emerging research hotspots and trends. The number of annual publications in this field of study has displayed a pattern of stabilization in the early years of the decade, followed by a notable increase in the later years and peaking in 2023 with 196 publications. Regarding publication volume and total citations, notable research contributions were observed from countries, institutions, and authors in North America, Western Europe, and China. Current research endeavors primarily focus on understanding the intervention mechanisms of neurodegenerative diseases through the ferroptosis pathway and exploring and identifying potential therapeutic targets.

**Conclusion:**

The study highlights key areas of interest and emerging trends in ferroptosis research on neurodegenerative diseases, offering valuable insights for further exploration and potential directions for diagnosing and treating such conditions.

## 1 Introduction

The progressive loss of neuronal structure and function or neuronal death in the brain and spinal cord characterizes neurodegenerative diseases (ND). Common examples include Alzheimer’s disease (AD), Parkinson’s disease (PD), Huntington’s disease (HD), amyotrophic lateral sclerosis, and multiple sclerosis (Dugger and Dickson, 2017; Agnello and Ciaccio, 2022; Temple, 2023). Iron tends to accumulate in specific brain regions with advancing age, triggering oxidative stress in cells and contributing to the onset of ND (Chen, 2019). Epidemiological studies have identified ferroptosis markers such as reduced GSH levels, iron deposition, and glutathione peroxidase 4 (GPX4) downregulation in cell and animal models of AD and PD (Cozzi et al., 2019). A study finds that ALOX5-mediated ferroptosis acts as a distinct cell death pathway upon oxidative stress in HD, it provides potential new targets for the treatment of HD (Song et al., 2023). Other researchers also explored the susceptibility to ferroptosis in fused in sarcoma-amyotrophic lateral sclerosis cell models, uncovering mitochondrial disturbances and heightened vulnerability to ferroptosis in cells containing amyotrophic lateral sclerosis-causing fused in sarcoma mutations. This result indicates that ferroptosis could play a substantial role in amyotrophic lateral sclerosis (Ismail et al., 2024). Additionally, One study highlights the benefits of dabrafenib in the treatment of multiple sclerosis by showing its ability to inhibit ferroptosis in microglia through the up-regulation of the Axl receptor. This mechanism ultimately helps to slow down the progression of multiple sclerosis (Liu et al., 2024a). Numerous research results indicate that inhibition of ferroptosis may be a potential strategy for treating ND.

**Figure 1:**
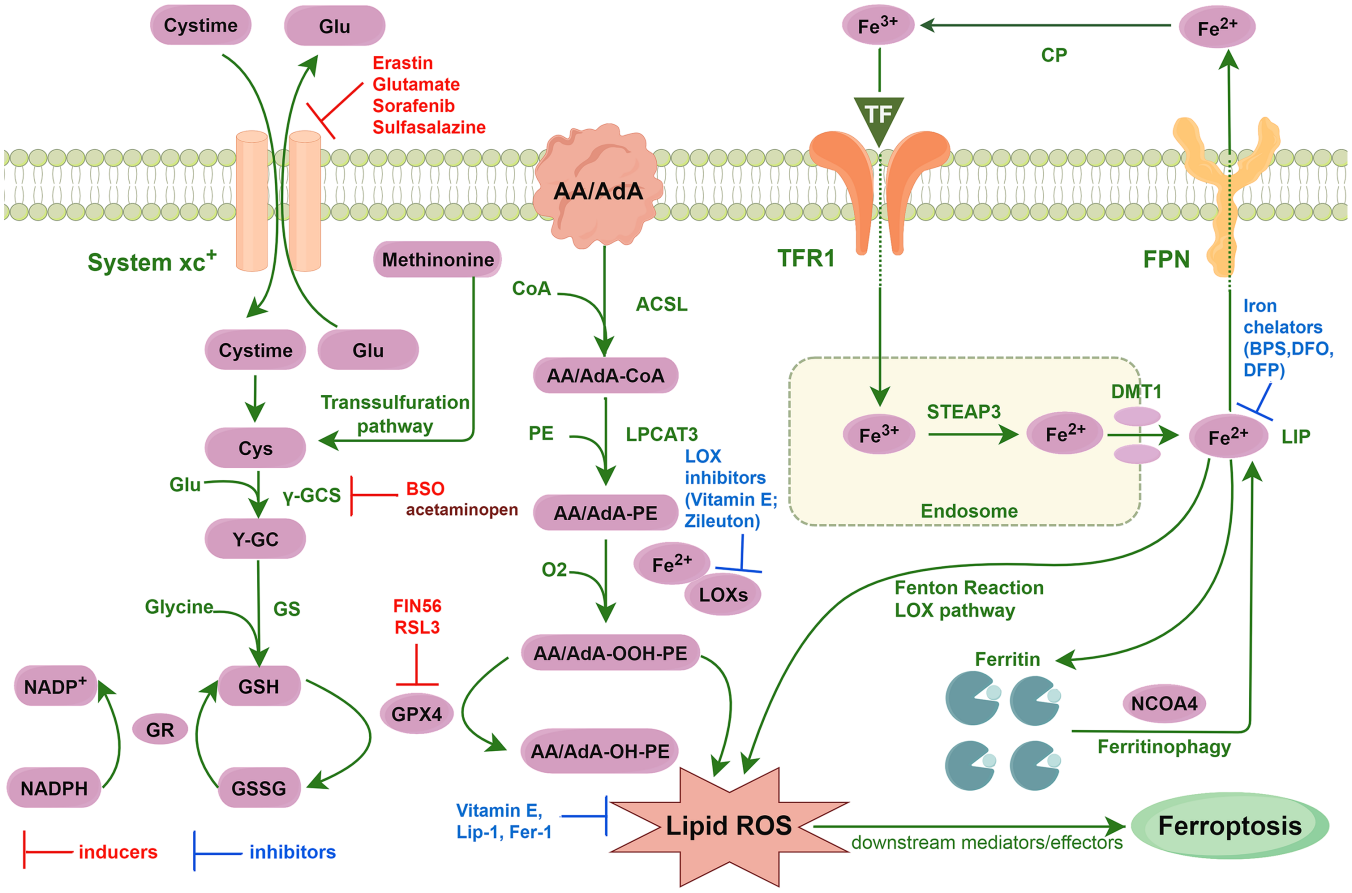
Core mechanisms of ferroptosis.

Ferroptosis is a newly identified type of iron-dependent cell death, distinguished by an accumulation of intracellular iron ions that disturb the equilibrium of the intracellular lipid peroxidation system, leading to lipid peroxidation and eventual cell demise (Nikseresht et al., 2019; Costa et al., 2023). In contrast to apoptosis, ferroptosis is an irregular and disordered way of cell death usually induced by external factors such as oxidative stress and drug impacts. Ferroptosis plays a significant role in a variety of diseases (Wang et al., 2023a), including nervous system diseases (Lei et al., 2020; Yao et al., 2021; Ou et al., 2022), cancers (Chen et al., 2021), lung diseases (Xu W. et al., 2021; Yu S. et al., 2021), cardiovascular diseases (Li N. et al., 2021; Yu Y. et al., 2021; Fang et al., 2023), liver diseases (Guo G. et al., 2023; Wang X. et al., 2023), and kidney diseases (Guo R. et al., 2023). The core mechanisms of ferroptosis (Figure 1) involve triggering cell death through the catalysis of lipid peroxidation of unsaturated fatty acids found in high levels on the cell membrane in the presence of divalent ferroptosis or ester oxygenase (Wu et al., 2021). The key enzyme responsible for this process is GPX4, which facilitates the conversion of phospholipid hydroperoxides into less harmful lipid alcohols, thereby safeguarding the cell from damage caused by lipid peroxidation. Inhibition or reduction of GPX4 activity enhances cellular susceptibility to ferroptosis (Bersuker et al., 2019; Xie et al., 2023). Additional regulatory factors of ferroptosis include disruptions in iron metabolism, accumulation of lipid peroxides, and imbalances in the antioxidant system. For instance, excessive iron intake or depleted iron reserves, along with heightened reactive oxygen species (ROS) generation through the Fenton reaction, can precipitate ferroptosis (Park and Chung, 2019). The mechanism of ferroptosis is intricate, involving the interplay of various factors and molecules. By modulating these factors, the sensitivity and response of cells to ferroptosis can be influenced. Pharmacological inhibition of ferroptosis by bioactive small-molecule compounds (ferroptosis inhibitors) could be effective for treatments of ND (Wang et al., 2023b). Aging is an unavoidable process of gradual deterioration of physiological functions caused by a combination of factors (Stockwell, 2022). As cellular senescence proceeds, the physiological functions of tissues and organs of the organism become progressively disturbed and decline, thereby increasing the risk of disease and death, such as ND, cancer and cardiovascular diseases (Goldsteins et al., 2022; Xu et al., 2024). At present, scholars have gradually discovered the intricate connection between ferroptosis and aging, and specific types of cellular senescence and aging-associated diseases are sometimes accompanied by features of cellular ferroptosis (Zheng et al., 2021).

Bibliometric analysis plays a vital role in assessing research performance and identifying influential papers within a specific field (Wang S. Q. et al., 2020). While citation analyses have been conducted in various areas of neuroscience, there is limited information on ferroptosis research related to ND, with only a few studies published. Citation counts significantly measure a study’s impact on a research field (Yi et al., 2022). Previous reviews have primarily relied on individual literature summaries and study extractions, which may not fully capture the spatial and temporal distribution of researchers, institutions, and journals. Additionally, visualizing the knowledge base’s internal structure and research focus has been challenging, with few systematic, comprehensive, and visual studies available. Current bibliometric analyses on ferroptosis primarily concentrate on specific diseases within the ND series, such as PD and AD. Researchers have identified pathogenesis and treatment (Chen et al., 2024) as key areas of interest in PD research, with ‘ferroptosis,’ ‘immunohistochemistry,’ ‘diagnosis,’ and ‘microenvironment’ being highlighted as frequent keywords (Lu et al., 2023). Similarly, in AD research, the focus lies on molecular mechanisms (Liu et al., 2024b), with ‘Ferroptosis in AD,’ ‘Pyrptosis in AD,’ and ‘Neoptosis in AD’ emerging as cutting-edge terms that indicate the present and future research directions in this field (Yeerlan et al., 2024). These studies have significantly contributed to the field of bibliometric analysis on ferroptosis and offer valuable research insights for further exploration in ND. Therefore, this study aims to perform a bibliometric analysis of papers published on ferroptosis in ND from 2014 to 2023, comprehensively analyzing the current research status, hot spots, and trends in this area. The goal is to identify journal publications, collaborators, keywords, and research trends that could enhance understanding of these diseases’ causes, mechanisms, and treatments. These findings offer valuable insights for future researchers conducting further research.

## 2 Materials and methods

### 2.1 Data source

This study utilized the Web of Science Core Collection (WoSCC) database, enriched by the Science Citation Index, as the primary data source. The WoSCC database is well-known for its comprehensive coverage, systematic methodology, and authoritative nature, spanning over 12,000 influential and high-quality journals globally. It is a commonly used resource in scientometric analyses and visualization of scientific literature across various research domains (Hassan et al., 2021; Ge et al., 2022). An advanced search was conducted in the WoSCC database using the search formula TS = (ferroptosis) AND TS = (Neurodegenerative Disease OR Neurologic Degenerative Disease OR Degenerative Neurologic Disease OR Nervous System Degenerative Diseases OR Neurodegenerative Disorder OR Neurologic Degenerative Condition OR Neurologic Degenerative Diseases OR Degenerative OR Neurologic Disorders OR Degenerative Neurologic Disorder) for a comprehensive investigation, focusing on articles and reviews in the English language published until December 2023. This search resulted in a total of 563 relevant documents.

### 2.2 Data import and merging

All data records from the WoSCC were extracted, including details such as annual research, countries/regions, funding agencies, source journals, institutions, authors, keywords, citations, impact factors, and Journal Citation Reports. Download the bibliographic record from the WoSCC database in Plain Text File format, selecting Record Content as Full Records and Cited References. Import the bibliographic record into NoteExpress and merge the entries in the fields manually after screening and verifying. We identified three situations where data needed to be merged and proposed solutions accordingly. These cases included: (1) full names or abbreviations of the same country name, such as different writing conventions for United States of America and USA; (2) different abbreviations of the same author’s name or variations in the order of the last name and the first name, which we solved by using ORCID information and author affiliation; and (3) different terms or expressions that refer to the same concept, for example, ND and neurodegenerative diseases will be merged into neurodegenerative diseases.

### 2.3 Data analysis and visualization

After data merging, the plain text was imported into Microsoft Excel 2019 and CiteSpace 6.2.R7 (Chen, 2004; Chen and Song, 2019) for analysis. Microsoft Excel was utilized to generate visualizations of the yearly publication volume, whereas CiteSpace was used to visualize collaborations among countries/regions, institutions, and authors. The analysis of cited literature primarily includes network maps, high-frequency co-cited literature, and keywords analysis comprising keywords co-occurrence analysis, keywords clustering analysis, and keywords citation burst analysis.

## 3 Results

### 3.1 Publication outputs and time trend

**Figure 2:**
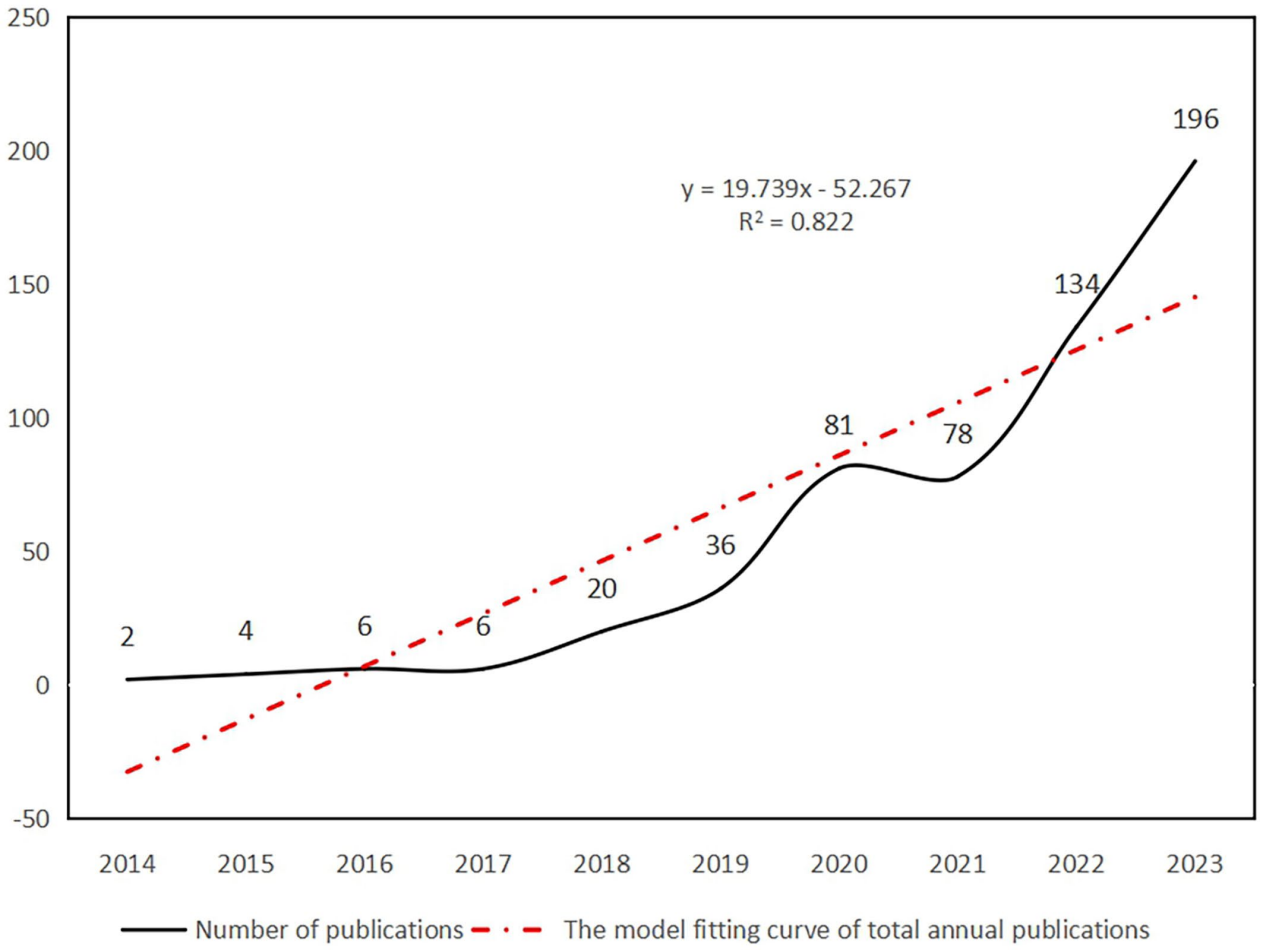
Number of publications and the model fitting curve of total annual publications.

A total of 563 documents on ferroptosis in ND research were published between 2014 and 2023. The publication trend exhibited stability in the initial years, followed by a notable surge in later years, as illustrated in Figure 2. From 2014 to 2017, the number of publications remained relatively low and consistent, with an annual range of 2–6 articles. Subsequently, there was a significant rise in publications from 2018 to 2020, with an average annual growth of 30 articles. Although there was a slight decline in 2021, the numbers remained comparable to 2020, indicating a sustained high activity level. A rapid growth trend is observed in 2022–2023, with an average increase of 79 articles per year, matching the total publications in 2021. Furthermore, linear regression analysis reveals a positive correlation between the annual total of published articles and the years (y = 19.739x - 52.267, R^2^ = 0.822). These results indicate a promising outlook for ongoing growth and advancement in this research field.

### 3.2 Distribution of journals

**Figure 3:**
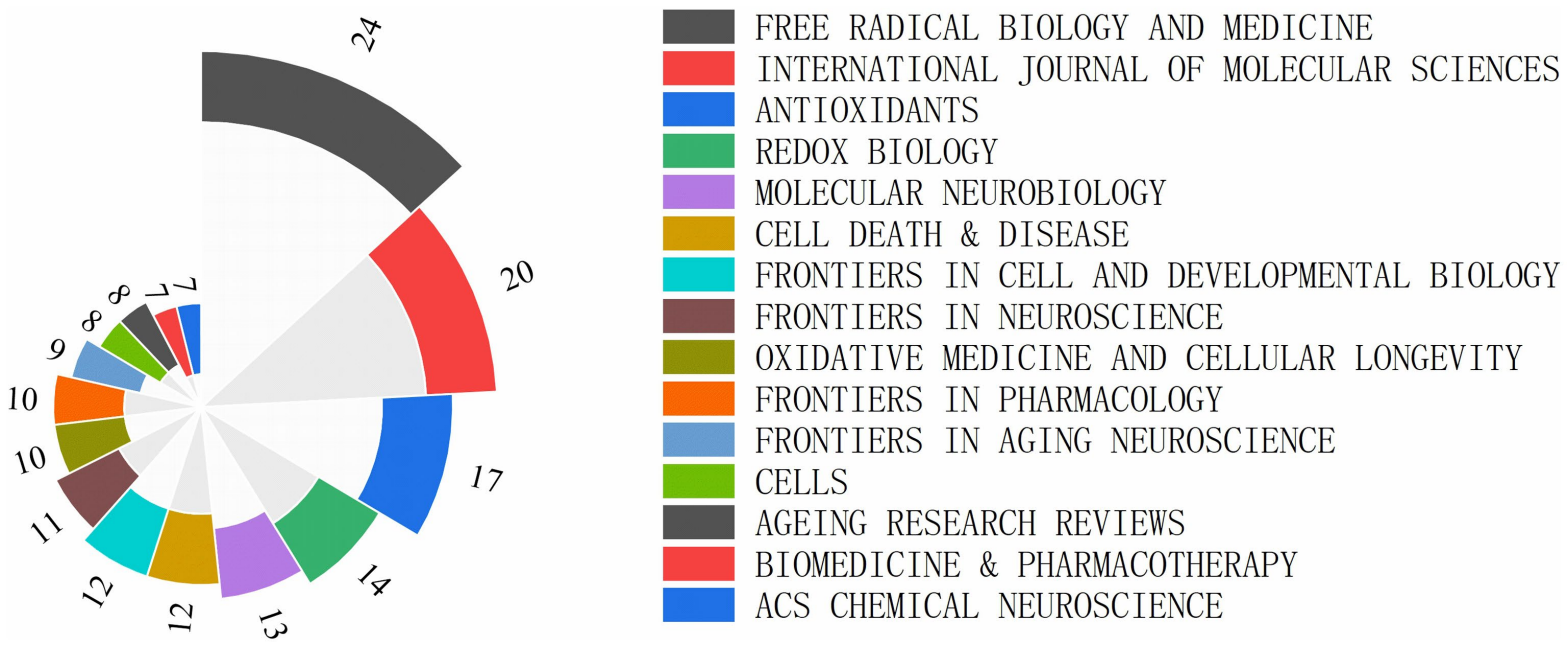
Top 15 journals of ferroptosis in ND research ranked by publication number.

The dataset consists of 563 journal articles from 280 different journals. The journal ‘FREE RADICAL BIOLOGY AND MEDICINE’ has the highest number of publications with 24 articles, followed by ‘INTERNATIONAL JOURNAL OF MOLECULAR SCIENCES’ with 20 articles. 90 journals have published more than 2 articles, making up 32.14% of the total number of journals and accounting for 69.8% of the total articles. The top 15 journals contribute to 32.33% of the total articles. These figures indicate a concentrated distribution of articles among journals, with those having high-impact factors publishing a significant number of articles. The top 15 journals by publication volume can be seen in Figure 3.

### 3.3 Analysis of countries/regions and institutions

**Figure 4:**
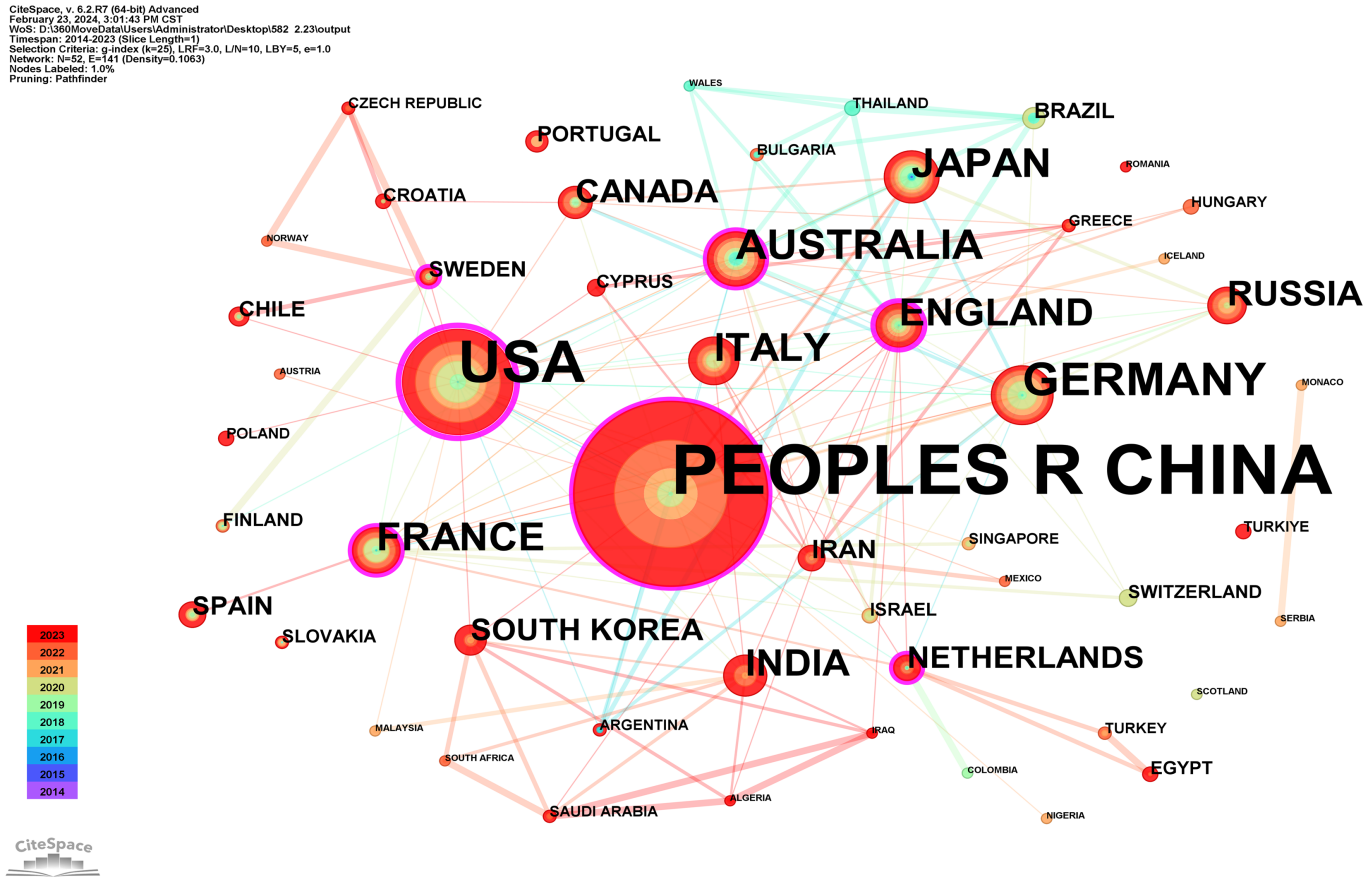
CiteSpace network visualization map of country/regions involved in ferroptosis of ND research.

A total of 52 countries participated in the studies analyzed, with 11 countries publishing more than 10 studies each. China emerged as the leading contributor with 43.6% of the studies, followed by the United States at 15.82% and Germany at 5.09%. The top 10 contributing countries are detailed in Table 1. CiteSpace analysis was utilized to create a visualization of country collaborations; as depicted in Figure 4, the network consists of 52 nodes and 141 links, illustrating the interconnected academic collaborations among high-producing countries. The top five countries identified are China, the United States, Germany, Australia, and Japan. Among these, the United States, England, and China emerge as the top three countries in terms of centrality, with values of 0.53, 0.37, and 0.18. Analysis based on publication numbers and centrality metrics highlights the United States, China, England, and Germany as the significant research powerhouses in this study.

**Table 1:** Top 10 countries and institutions in the field of ferroptosis in ND among 563 included studies (2014–2023).

Rank	Country	Count	Centrality	Institute (Country)	Count	Centrality
1	CHINA	317	0.18	University of Melbourne (Australia)	21	0.12
2	USA	115	0.53	Helmholtz Association (Germany)	21	0.04
3	GERMANY	37	0.08	Helmholtz-Center Munich - German Research Center for Environmental Health (Germany)	18	0.03
4	AUSTRALIA	25	0.15	Florey Institute of Neuroscience & Mental Health (Australia)	17	0.12
5	JAPAN	24	0	University of Texas System (USA)	17	0.12
6	ITALY	22	0.02	Institut National de la Sante et de la Recherche Medicale (France)	16	0.12
7	FRANCE	21	0.15	Harvard University (USA)	14	0.07
8	ENGLAND	20	0.37	China Medical University (China)	14	0.01
9	INDIA	17	0.08	Shanghai Jiao Tong University (China)	13	0.07
10	RUSSIA	14	0	Huazhong University of Science & Technology (China)	13	0.01

**Figure 5:**
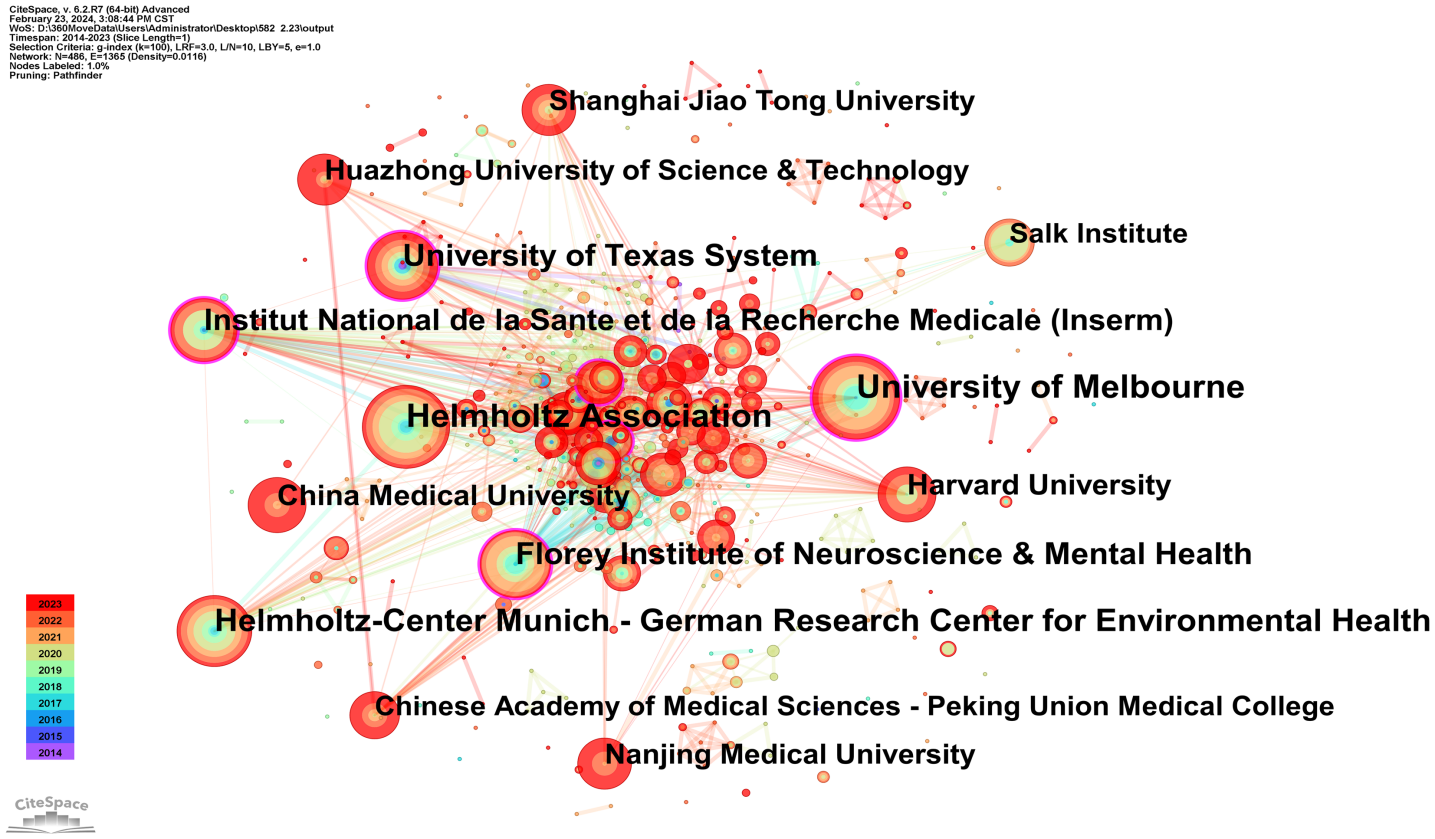
CiteSpace network visualization map of institutions involved in ferroptosis of ND research.

A total of 486 institutions participated in publishing research papers, with 59 institutions (12.14%) contributing more than 5 papers. The top 10 institutions, detailed in Table 1, each produced at least 13 papers. The University of Melbourne and the Helmholtz Association led with 21 papers each. Collaborative institution mapping results, illustrated in Figure 5, showcased 486 nodes and 1,365 links, indicating cooperative solid relationships. The University of Melbourne, Helmholtz Association, Helmholtz-Center Munich-German Research Center for Environmental Health, Florey Institute of Neuroscience & Mental Health, and the University of Texas System emerged as the top five institutions in collaboration. Noteworthy is the joint top position in centrality rankings held by the University of Melbourne, Florey Institute of Neuroscience & Mental Health, University of Texas System, and Institut National de la Sante et de la Recherche Medicale.

### 3.4 Analysis of authors and co-cited representative literature

**Figure 6:**
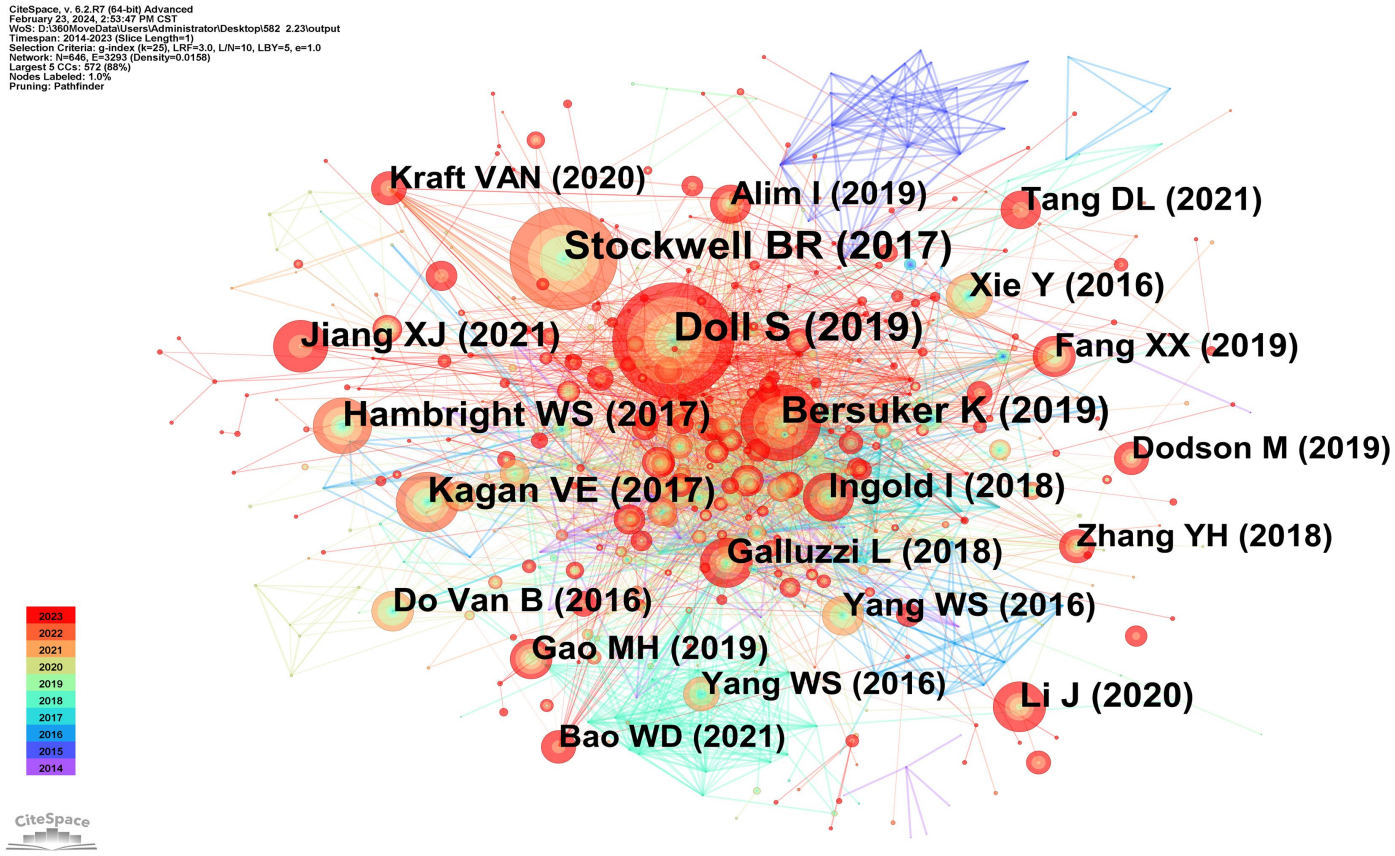
CiteSpace network visualization map of co-cited references involved in ferroptosis of ND research.

There are 803 authors involved in Ferroptosis in ND. The top 10 most active authors and their related information are shown in Table 2. The top 10 authors collectively published 78 articles. Conrad Marcus published 14 papers and ranked first among all authors, followed by Maher Pamela (*n* = 10) and Hirata Yoko (*n* = 9). These top 10 authors were affiliated with 8 different research institutions. Table 3 displays the top 10 most cited original articles on ferroptosis in ND research. Nature Chemical Biology and Cell have had a significant scientific impact on researchers and scholars in this field, with nearly half of the top 10 highly cited original articles being published in these journals. All of the top 10 publications have collectively received over 1,030 citations. The study by Doll S et al., published in Nature Chemical Biology, is the most cited article with 182 citations (Doll et al., 2017). Figure 6 presents the results of the CiteSpace analysis of literature co-citations, which includes 646 nodes and 3,293 links. Both nodes and links show the richness of co-citations.

**Table 2:** Top 10 authors who published literature on ferroptosis in ND among 563 included studies (2014–2023).

Rank	Author	Institute	Count	Centrality
1	Conrad, Marcus	Helmholtz Zentrum Munchen	14	0.00
2	Maher, Pamela	Salk Institute for Biological Studies	10	0.00
3	Hirata, Yoko	Gifu University	9	0.00
4	Devos, David	Lille University	7	0.01
5	Furuta, Kyoji	Gifu University	7	0.00
6	Currais, Antonio	Salk Institute for Biological Studies	7	0.00
7	Bush, Ashley I	University of Melbourne	6	0.02
8	Ayton, Scott	University of Melbourne	6	0.01
9	Tang, Daolin	Guangzhou Medical University	6	0.00
10	Stockwell, Brent R.	Columbia University	6	0.00

**Table 3:** Top 10 co-citation representative literature of ferroptosis in ND among the 563 articles included (2014–2023).

Rank	Cited number	Title	Type	Year	Centrality	Journal	JCR (2022)	IF (2022)	Reference
1	182	ACSL4 dictates ferroptosis sensitivity by shaping cellular lipid composition	Article	2017	0.04	Nature Chemical Biology	Q1	14.8	Doll et al. (2017)
2	161	Ferroptosis: A Regulated Cell Death Nexus Linking Metabolism, Redox Biology, and Disease	Review	2017	0.00	Cell	Q1	64.5	Stockwell et al. (2017)
3	120	The CoQ oxidoreductase FSP1 acts parallel to GPX4 to inhibit ferroptosis	Article	2019	0.02	Nature	Q1	64.8	Bersuker et al. (2019)
4	94	Oxidized arachidonic and adrenic PEs navigate cells to ferroptosis	Article	2017	0.02	Nature Chemical Biology	Q1	14.8	Kagan et al. (2017)
5	88	Ablation of ferroptosis regulator glutathione peroxidase 4 in forebrain neurons promotes cognitive impairment and neurodegeneration	Article	2017	0.01	Redox biology	Q1	11.4	Hambright et al. (2017)
6	82	Ferroptosis: mechanisms, biology and role in disease	Review	2021	0.00	Nature reviews Molecular cell biology	Q1	112.7	Jiang et al. (2021)
7	79	Ferroptosis: past, present and future	Review	2020	0.01	Cell Death & Disease	Q1	9.0	Li et al. (2020)
8	78	Molecular mechanisms of cell death: recommendations of the Nomenclature Committee on Cell Death 2018	Review	2018	0.09	Cell Death and Differentiation	Q1	12.4	Galluzzi et al. (2018)
9	76	Selenium Utilization by GPX4 Is Required to Prevent Hydroperoxide-Induced Ferroptosis	Article	2018	0.04	Cell	Q1	64.5	Ingold et al. (2018)
10	70	Ferroptosis: process and function	Review	2016	0.00	Cell Death and Differentiation	Q1	12.4	Xie et al. (2016)

^*^IF, impact factor; *JCR, Category Quartile of Journal Citation Report.

### 3.5 Keyword analysis

#### 3.5.1 Keywords co-occurrence analysis

Keywords related to ferroptosis in ND research papers were identified and analyzed using CiteSpace software. The keywords were analyzed over The publication period of 2014–2023. The Top five keywords were oxidative stress (*n* = 229), cell death (*n* = 206), lipid peroxidation (*n* = 150), AD (*n* = 115), and PD (*n* = 103). Larger values of centrality represent more cooperation of The node with other nodes. Centrality analysis revealed that cancer cells (*n* = 0.16), activation (*n* = 0.12), ferroptosis (*n* = 0.11), cell death (*n* = 0.09), and mechanisms (*n* = 0.09) exhibited high centrality and emerged as the most influential keywords.

#### 3.5.2 Keywords clusters analysis

**Figure 7:**
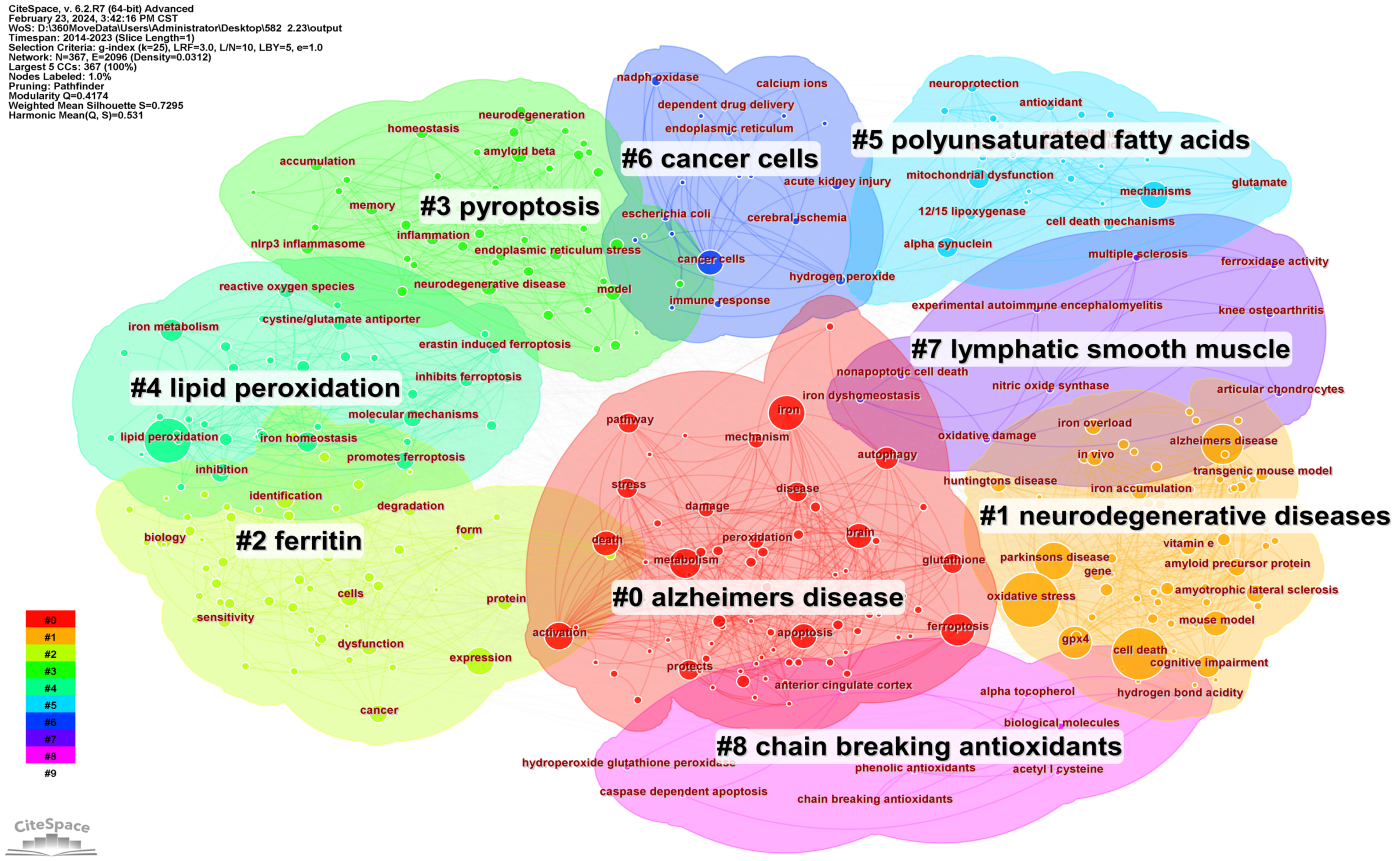
CiteSpace network visualization map of keywords clusters analysis involved in ferroptosis of ND research.

The keyword clustering graph reflects the structural features between clusters, highlighting their key nodes and essential connections (Qin et al., 2020). Based on the results of the co-occurrence analysis, a keyword clustering network map was generated with LLR. The network map has a rich clustering structure, and the structure is persuasive, as indicated by the Modularity Q= 0.4174 > 0.3 and Mean Silhouette= 0.7295 > 0.5 of the keyword clustering map, signifying that the clustering structure is clear and reasonable, with high credibility. As is shown in Figure 7, the top 9 clustered tag groups (#0-8), examined and filtered using clustered keywords, represent a general research framework for ferroptosis in ND research. Among the nine keyword clusters, the research topics can be categorized into three groups: (#0), (#1), and (#7) form the first group, mainly related to the study of diseases caused by ferroptosis; (#2), (#5), and (#6) form the second group, mainly related to the study of the effects of ferroptosis on body tissues and cells; (#3), (#4), and (#8) form the third group, mainly involved in the study of the primary mechanism of ferroptosis in ND. The number of labels in the keyword clusters is inversely proportional to the size of the clusters, with the most prominent clustered label being (#0) AD (*n*=76), indicating that AD is the primary disease in ferroptosis in ND research.

#### 3.5.3 Keywords citation burst analysis

**Figure 8:**
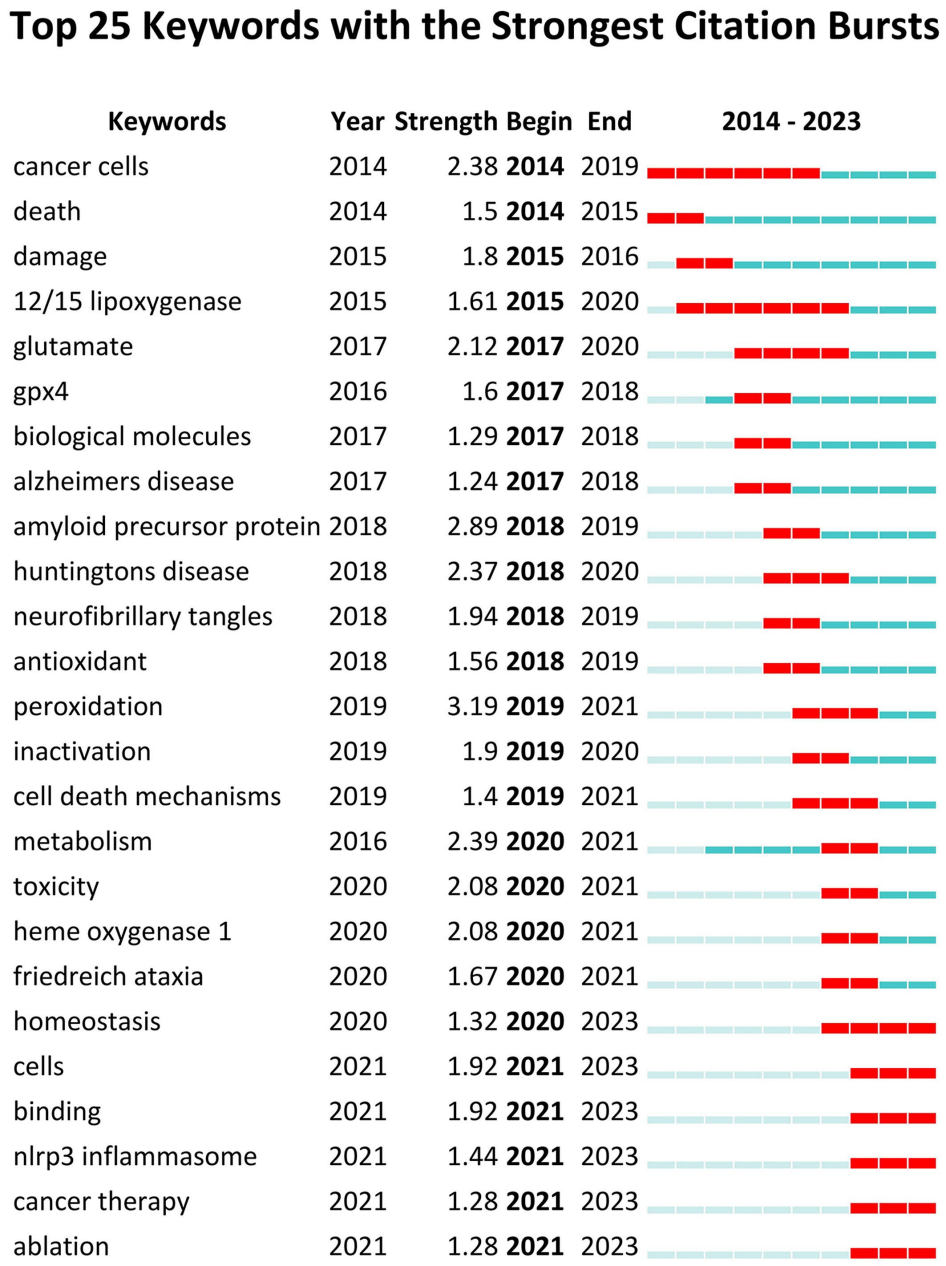
Keywords citation bursts analysis of related literature. The red line shows the time frame during which the keyword bursts were discovered, while the blue line shows the time interval.

The analysis of keyword citation bursts can reveal the research trends within a specific timeframe (Wang Z. et al., 2020). Based on keyword co-occurrence analysis, Keyword citation burst analysis was utilized to determine the top 25 cited keywords, see Figure 8. In the Keywords citation burst analysis, “Begin” and “End” indicate the time of the burst. “Strength” means the strength of the burst, representing credibility over time. By focusing on keywords with a high burst rate, researchers can uncover the current hot topics in the field.

The Keywords citation burst analysis reveals three phases of results. The first phase, from 2014 to 2017, highlights keywords like ‘cancer cells, death, injury, 12/15 lipoxygenase, glutamate, gpx4, biomolecules, AD’, focusing on physiological changes and disease effects. For instance, the proliferation of cancer cells was linked to ferroptosis, which was also identified as a pathogenic mechanism in AD. The second phase, spanning 2017 to 2020, explores keywords such as ‘amyloid precursor protein, HD, neurofibrillary tangles, antioxidant, peroxidation, inactivation, cell death mechanisms, metabolism, toxicity, heme oxygenase 1, Friedreich’s ataxia,’ emphasizing the impact of ferroptosis on ND. Lastly, the third phase, from 2020 to 2023, concentrates on ‘homeostasis, cellular, binding, NLRP3 inflammasome, cancer therapy, ablation,’ indicating a sustained focus on mechanism and pathway studies. This phase also underscores the increasing significance of inhibition of ferroptosis for disease treatment. The keywords from the third phase remain relevant today and represent emerging trends and future research directions.

## 4 Discussion

### 4.1 General information

In the past decade, there has been a notable increase in scholarly interest and research on ferroptosis in ND, leading to a growing number of related studies annually. This bibliometric analysis employs CiteSpace to examine and illustrate 563 papers on the subject to identify critical research hotspots and trends. The evolution of research activity and output in publications related to this topic can be observed in three distinct phases. Before 2018, a relatively consistent and limited number of papers were published. In 2012, the discovery of a small molecule compound called erastin revolutionized the field by affecting a wide range of cells, including neurons and tumor cells. Erastin enhances the process of peroxide accumulation during Fe2^+^ synthesis, leading to intracellular mitochondrial atrophy and increased membrane density. This unique mechanism results in a distinctive form of cell death known as ferroptosis, setting it apart from other forms of cell death (Li et al., 2020). Initially, research on ferroptosis was limited. However, from 2018 to 2021, there was a substantial increase in the number of papers, indicating a broadening scope and depth of research. Post-2021, there has been a rapid growth trend, with an average annual increase of approximately 79 articles. This trend underscores the immense research potential of ferroptosis in ND, drawing increasing attention from scholars (Reichert et al., 2020; Xu et al., 2023). Consequently, it is foreseeable that this research area will continue to gain traction in the future.

Among the top 10 countries publishing papers in this research field, 9 are developed, while China is the sole developing country. Despite China having the highest number of publications, its low centrality score of 0.18 suggests limited connections with developed countries in the field, indicating a lack of international cooperation. The United States, Germany, the United Kingdom, and Australia dominate this area. Analysis of the institutional cooperation network reveals that national institutions in Australia, the United States, and Germany, such as the University of Melbourne, Helmholtz Association, Helmholtz-Center Munich - German Research Center for Environmental Health, Florey Institute of Neuroscience & Mental Health, and the University of Texas System, are leading in research on ferroptosis in ND. The most active and prominent institutions are predominantly renowned universities from developed countries with abundant academic resources, with China following closely behind. There is an imbalance in the exchange of academic resources between developing and developed countries. This disparity may be attributed to financial constraints and insufficient attention in developing countries, resulting in delayed research initiation and a lack of high-quality research outcomes.

The distribution of author clusters exhibits similarities to the distribution of country and institutional clusters. The top 10 authors listed in Table 2 are predominantly associated with research institutions in developed countries. Among these top 10 authors, Conrad Marcus, Maher Pamela, and Hirata Yoko emerge as the leading researchers in this particular field. Conrad Marcus has proposed that ferroptosis plays a significant role in the development of organ injuries and degenerative pathologies. He has also suggested that the manipulation of ferroptosis, through both its induction and inhibition, holds promise in treating drug-resistant cancers, ischaemic organ injuries, and other degenerative diseases characterized by high levels of lipid peroxidation (Jiang et al., 2021). Maher Pamela and her colleagues have emphasized the involvement of the oxytosis/ferroptosis is regulated with cell death pathway in aging and ND and the AMPK/ACC1 pathway in cell death induced by an oxytosis/ferroptosis inducer (Currais et al., 2022). Additionally, Hirata Yoko and his team discovered that N,N-dimethylaniline derivatives had been identified as potent inhibitors of ferroptosis. Despite not forming a chelating structure with Fe2^+^, N,N-dimethylaniline derivatives are capable of creating stable monodentate complexes with hydrated ferrous ions, with the assistance of aliphatic tertiary amine molecules to stabilize the complexes. Those discoveries could potentially aid in developing more effective lysosomal ferroptosis inhibitors for treating ND (Hirata et al., 2023).

Analysis of publication output and citations reveals that approximately one-third of the papers are published in the top 15 journals. Notably, ‘Nature Chemical Biology’ and ‘Cell’ have had a significant scientific impact on researchers and scholars in the field. For instance, ‘Nature Chemical Biology’, a journal under the NATURE PORTFOLIO, boasts an IF of 14.8 in 2022. The journal covers diverse research on the role of ferroptosis in ND, including lipid metabolism, autophagy, and mitochondria, which significantly advances people’s understanding of this field. An article in the journal investigated the relationship between the structure, activity, and distribution of compounds that modulate ferroptosis using fluorescence and stimulated Raman scattering imaging. The study found that lipid peroxidation triggering ferroptosis occurs across different subcellular membranes, with the endoplasmic reticulum membrane being identified as the primary site. The research revealed a sequential pattern of membrane peroxidation during iron metabolism, starting with accumulation on the endoplasmic reticulum membrane before spreading to the plasma membrane. By targeting inhibitors and inducers specifically at the endoplasmic reticulum, it may be possible to precisely regulate lipid peroxidation dynamics in cells undergoing ferroptosis. This targeted approach shows promise as a potential therapeutic strategy for drug-resistant cancers and could have implications for the pathogenesis of certain degenerative diseases (Von Krusenstiern et al., 2023). Furthermore, other prestigious journals like ‘Nature’, ‘Nature Reviews Molecular Cell Biology’, and ‘Cell Death and Differentiation’ have also made valuable contributions to the field of ferroptosis to ND research.

### 4.2 Important research findings

Based on a literature citation analysis, the paper ‘ACSL4 dictates ferroptosis sensitivity by shaping cellular lipid composition’ by Doll S et al. published in ‘Nature Chemical Biology’ in 2017 (Doll et al., 2017) has received the highest number of citations. The study highlights the crucial role of CoA synthetase long-chain family member 4 (ACSL4) in driving ferroptosis. GPX4-ACSL4 double-knockout cells displayed significant resistance to ferroptosis. Targeting ACSL4 with thiazolidinediones, a type of antidiabetic compound, reduced tissue damage in a mouse model of ferroptosis, suggesting that inhibiting ACSL4 could be a promising therapeutic approach for ferroptosis-related diseases. The second most cited work is a review by Stockwell BR et al. published in Cell in 2017 (Stockwell et al., 2017), which underscores that recent research indicates ferroptosis is triggered by integrating polyunsaturated fatty acids into cellular membranes. Sensitivity to ferroptosis is intricately linked to various biological processes, including amino acid, iron, and polyunsaturated fatty acid metabolism and the synthesis of glutathione, phospholipids, and coenzyme Q10. Ferroptosis has been implicated in pathological cell death in conditions such as ND, cancer progression, stroke, intracerebral hemorrhage, and traumatic brain injury.

In addition, in an article published in Nature Chemical Biology, Kagan et al. (2017) demonstrated that ferroptosis is characterized by a highly organized oxygenation center. They discovered that inhibiting ACSL4 can prevent the esterification of arachidonic acid or adrenic acid into phosphatidylethanolamine, serving as a specific pathway to rescue cells from ferroptotic cell death. Lipoxygenase was identified as the enzyme responsible for generating doubly and triply-oxygenated-diacylated phosphatidylethanolamine species, which signal cell death. Additionally, tocopherols and tocotrienols were found to suppress lipoxygenase activity and protect cells from ferroptosis, indicating a potential physiological role for vitamin E in maintaining cellular homeostasis. The authors propose that targeting this oxidative phosphatidylethanolamine death pathway could be a promising approach for drug development (Kagan et al., 2017).

### 4.3 Research hotspots and frontiers

Keywords play a crucial role in identifying emerging trends and guiding future research directions (Synnestvedt et al., 2005). In this instance, keywords citation burst analysis was conducted using CiteSpace. Five research frontier keywords burst from 2021. We summarize the existing research results and provide insights into the future research direction hotspots of ferroptosis on ND.

#### 4.3.1 Cells

Ferroptosis is a unique type of regulated cell death characterized by the lethal buildup of lipid peroxides in plasma membranes (Martinez et al., 2020). This process is intricately linked to various biological pathways and has been implicated in the pathogenesis of cancer and ND. These discoveries have paved the way for developing innovative cytoprotective approaches aimed at safeguarding cells in conditions such as neurodegenerative, hematologic, and cardiovascular disorders through inhibiting ferroptosis (Qiu et al., 2020). The study of microglia has been increasingly emphasized by scholars, and research on microglia ferroptosis in ND has been carried out. A study found that human-induced pluripotent stem cell-derived microglia when cultivated in a tri-culture system, demonstrate heightened reactivity to iron and are prone to ferroptosis. Excessive iron levels cause a notable shift in the transcriptional profile of microglia, resembling a gene expression pattern seen in postmortem brain microglia of PD patients. This microglial reaction contributes to neurodegeneration, as evidenced by the delayed onset of iron-induced neurotoxicity upon microglia removal from the tri-culture setup. These results underscore the critical role of microglial iron overload and ferroptosis in the neurodegenerative process (Ryan et al., 2023).

Aberrations in programmed cell death signaling pathways, such as apoptosis, necroptosis, pyroptosis, ferroptosis, autophagy-associated cell death, and unprogrammed necrosis, are observed in the development of various neurological disorders. These mechanisms of cell death can be initiated by cellular stress and inflammatory reactions. The dysregulated activation of programmed cell death pathways is a common feature in neurodegenerative conditions, resulting in the unwanted loss of neuronal cells and their functionality (Moujalled et al., 2021). Mitochondria are pivotal in the process of apoptotic cell death, with mitochondrial outer membrane permeabilization (MOMP) typically leading to cell death (Vringer and Tait, 2023). MOMP triggers a range of pro-inflammatory signaling pathways, and recent research indicating cell survival after MOMP suggests that mitochondria-derived signaling from pro-apoptotic triggers may have non-lethal functions. Targeting MOMP to modulate cell death shows great promise for therapeutic interventions in ND (Bock and Tait, 2020). Research has been conducted on the cellular level of ferroptosis in the context of drug treatment for ND. Experimental animal studies have demonstrated that salidroside exhibits neuroprotective effects by inhibiting neuronal ferroptosis in Amyloid beta peptide (Aβ)1-42-induced AD mice and Glu-injured HT22 cells. This neuroprotective mechanism is associated with the activation of the Nrf2/HO1 signaling pathway. This area of research shows promise for future exploration (Yang et al., 2022).

#### 4.3.2 Binding

GPX4 is identified as a crucial regulator of ferroptosis. Autoantibodies and interferon-α found in the serum have been shown to trigger neutrophil ferroptosis by increasing the binding of the transcriptional repressor CREMα to the GPX4 promoter. This results in reduced expression of GPX4 and an increase in lipid-ROS (Li P. et al., 2021). High mobility group box 1 (HMGB1) is a nonhistone nuclear protein known for its role as a DNA chaperone, maintaining chromosome structure and function. Additionally, HMGB1 can induce autophagy by interacting with the BECN1 protein. The secretion and release of HMGB1 are regulated by various factors, such as posttranslational modifications like phosphorylation and methylation, as well as cell death mechanisms like apoptosis, necroptosis, and ferroptosis (Chen et al., 2022).

The RNA-binding protein ZFP36 ring finger protein is essential in regulating ferroptosis in hepatic stellate cells (HSCs). Treatment with erastin and sorafenib in mice helped alleviate murine liver fibrosis by promoting HSC ferroptosis. Overexpression of ZFP36, specifically in HSCs, hindered the induction of HSC ferroptosis by erastin or sorafenib. In human HSCs, sorafenib treatment alone resulted in ZFP36 downregulation, activation of ferritinophagy, and induction of ferroptosis. These findings shed light on new molecular mechanisms and signaling pathways involved in ferroptosis (Zhang et al., 2020). Another study (Zhang et al., 2021) suggests that ferroptosis is a tightly regulated process suppressing tumors. The RNA-binding protein RBMS1 plays a role in lung cancer development by facilitating the evasion of ferroptosis, making it a crucial regulator of this process. Inhibiting RBMS1 led to decreased translation of SLC7A11, resulting in reduced cystine uptake mediated by SLC7A11 and ultimately promoting ferroptosis. Exploring the role of RNA-binding proteins in disease development through mediating ferroptosis evasion could be a promising area for further research.

#### 4.3.3 NLRP3 inflammasome

Deferoxamine, an iron chelator, has demonstrated the ability to inhibit neuron degeneration by reducing the accumulation of iron, lipid peroxides, and ROS and modulating the expression of ferroptosis-related indicators. Furthermore, Deferoxamine has shown potential in reducing NLRP3 activation through the ROS/NF-κB pathway, influencing microglial polarization, decreasing neutrophil and macrophage infiltration, and inhibiting the release of inflammatory factors following traumatic brain injury. Moreover, Deferoxamine may also attenuate the activation of neurotoxic responsive astrocytes (Jia et al., 2023). It has been proposed that therapeutic interventions for neuropsychiatric disorders may benefit from targeting not only oxidative stress and inflammatory processes in general but also specific factors such as TNF-α, PARP-1, NLRP3 inflammasome, and RIP3. Activation of these factors can lead to peripheral inflammation and neuroinflammation, even in the absence of cell death, highlighting their potential as treatment targets (Morris et al., 2018).

Aβ has been identified as a key contributor to AD. In a study involving APP/PS1 mice, treatment with non-toxic tetrahydroxy stilbene glycoside (TSG) demonstrated a dose-dependent protection against Aβ-induced neuronal cell death by modulating ferroptosis-related proteins and enzymes. TSG was found to enhance the activation of GSH/GPX4/ROS and Keap1/Nrf2/ARE signaling pathways. Additionally, TSG administration led to a decrease in markers associated with ferroptosis, such as lipid peroxidation and neuroinflammation markers like NLRP3 and ACSL4 (Gao et al., 2021). Elevated expression of Heme oxygenase-1 in ND has been linked to the accumulation of neurotoxic ferric iron deposits. In mouse models, inflammation-induced stimulation leads to changes in various iron-related metabolic proteins, ultimately causing an upsurge in ferroptosis, iron deposition, and oxidative stress. Additionally, microglia demonstrate a primed phenotype with heightened levels of inflammatory markers, including iNOS, TNF-α, IL-1β, and NLRP3 (Fernández-Mendívil et al., 2021). Exploring the NLRP3 inflammasome pathway and its molecular interactions may reveal additional potential avenues for the development of inflammasome-targeting drugs.

#### 4.3.4 Cancer therapy

Ferroptosis induction has recently gained attention as a promising approach to cancer treatment. Elevated iron levels within cells lead to increased production of ROS. Molecules that induce ferroptosis enhance ROS production and suppress the antioxidant defense system, thereby promoting ferroptosis in cancer cells (Maru et al., 2022). Cancer cells accumulate elevated levels of iron and ROS to enhance their metabolic activity and proliferation. Notably, the metabolic reprogramming of cancer cells is frequently linked with increased vulnerability to ferroptosis. This indicates that ferroptosis could serve as an adaptive reaction to metabolic dysregulation and potentially offer a novel approach to eliminating cancerous cells (Battaglia et al., 2020).

RSL3 and other small-molecule GPX4 inhibitors have been shown to induce ferroptosis in both cultured cancer cells and tumor xenografts in mice. Likewise, erastin and other system Xc- inhibitors can reduce intracellular glutathione levels necessary for GPX4 activity, resulting in lipid peroxidation and ferroptosis. Given that therapy-resistant cancer cells are particularly vulnerable to GPX4-targeted treatments, the potential of ferroptosis-inducing agents to enhance existing cancer therapies is promising (Wang L. et al., 2022). Glioblastoma is recognized as the predominant malignant tumor affecting the brain. The complexity of this cancer poses challenges in treatment, attributed to its significant heterogeneity and the presence of an immunosuppressive microenvironment. Recent research indicates that directing interventions toward ferroptosis could serve as a promising approach to addressing resistance to conventional tumor therapies and immune evasion strategies (Zhuo et al., 2022). The integration of ferroptosis-targeted therapies with existing treatments holds the potential to enhance the management of cancer.

#### 4.3.5 Ablation

In various neurodegenerative disorders, the intracellular accumulation of TAU aggregates is a defining feature. When TAU was conditionally removed from excitatory neurons, it led to a decrease in epilepsy and the overactivation of the PI3K/AKT pathway (Shao et al., 2022). Using CRISPR to introduce point mutations and express human tau in AD model mice, it was demonstrated that the interaction between specific sites regulates both normal physiological phosphorylation and the development of amyloid-associated cognitive impairments. Furthermore, a combined approach targeting key phosphorylation sites and p38α synergistically ablated hyperphosphorylation (Stefanoska et al., 2022). Additionally, Shank3, a protein primarily found in the heart, plays a role in regulating age-related ND. Removal of Shank3 was found to increase the process of mitophagy, decrease the production of harmful superoxide from mitochondria, reduce cell death, and protect against heart dysfunction in older individuals. When aging heart cells treated with D-galactose were studied, it was observed that mitophagy decreased while Shank3 expression significantly increased. Knocking down Shank3 resulted in the restoration of mitophagy, leading to improved health of mitochondria, reduced oxidative stress within mitochondria, and decreased cell death. Conversely, overexpression of Shank3 mimicked the inhibitory effects of D-galactose on mitophagy and caused dysfunction in mitochondria (Wang Y. et al., 2022). Another study reported that aconitate decarboxylase 1 ablation abates Tumor-infiltrating neutrophils infiltration, constrains metastasis, and bolsters antitumor T cell immunity (Zhao et al., 2023). Previously, we mentioned GPX4’s research on cancer; in a new study, it was found that the loss of GPX4 leads to an excessive buildup of lipid peroxides and ferroptosis in Treg cells when T cell receptor/CD28 co-stimulation occurs. Additionally, Treg-specific ablation of GPX4 inhibits tumor growth and enhances the body’s ability to fight against tumors (Xu C. et al., 2021).

A recent study delved into the concept of ‘ablation’ within the realm of treatment technology (Pfannenstiel et al., 2022). Microwave ablation (MWA) is gaining attention as an alternative method for treating unresectable tumors. Studies suggest that MWA may impact the process of ferroptosis within tumors, potentially regulating cancer cell ferroptosis. This mechanism could play a role in the tumor-killing effects of MWA (Bozaykut et al., 2016). Research indicates that inducing ferroptosis is a promising therapy for cancer treatment, suggesting that combining MWA with ferroptosis inducers could be a novel approach in tumor therapy (Gai et al., 2020; Yu et al., 2023). In conclusion, the specific ablation of TAU, Shank3, and GPX4, along with utilizing MWA technology, presents new opportunities for studying ND and cancer induced by ferroptosis.

### 4.4 Limitations

This study utilized CiteSpace 6.2.R7 software to visualize and analyze the literature on ferroptosis in ND within the WoSCC database. However, several limitations were identified. Firstly, disparities in economic strength and population size among countries may influence research progress in this field, potentially leading to bias. Secondly, only English literature from the WoSCC database was included in the visualization analysis, suggesting that future studies should consider incorporating additional databases such as CNKI and Scopus. Lastly, since citations are expected to peak gradually 3–10 years after publication, the current analysis may not highlight recently published articles.

## 5 Conclusion

Research on ferroptosis for ND is still in its early stages of development. Based on the current global trend, there is expected to be a significant increase in publications on this topic and the number of researchers actively involved. Noteworthy advancements have been achieved in ferroptosis research related to ND, particularly in China, Europe, and the United States, with Europe and the United States emerging as leaders in this field. Existing literature can be broadly classified into two main categories: ‘mechanism studies’ and ‘treatment and effects’. Future research is likely to focus more on areas such as cells, binding, nlrp3 inflammasome, cancer therapy, and ablation, indicating potential new directions for investigation.

## Author contributions

YL: Data curation, Funding acquisition, Investigation, Project administration, Resources, Software, Supervision, Validation, Visualization, Writing – original draft, Writing – review & editing. DF: Methodology, Software, Validation, Visualization, Writing – review & editing. LS: Data curation, Investigation, Project administration, Visualization, Writing – review & editing. Y-jW: Formal analysis, Visualization, Writing – review & editing. LY: Validation, Writing – review & editing. Y-qL: Investigation, Validation, Writing – review & editing. J-yT: Funding acquisition, Supervision, Writing – review & editing.
